# Mass Spectrometry Imaging Reveals Neutrophil Defensins as Additional Biomarkers for Anti-PD-(L)1 Immunotherapy Response in NSCLC Patients

**DOI:** 10.3390/cancers12040863

**Published:** 2020-04-02

**Authors:** Eline Berghmans, Julie Jacobs, Christophe Deben, Christophe Hermans, Glenn Broeckx, Evelien Smits, Evelyne Maes, Jo Raskin, Patrick Pauwels, Geert Baggerman

**Affiliations:** 1 Centre for Proteomics, University of Antwerp, 2020 Antwerpen, Belgium; eline.berghmans@vito.be; 2 Health Unit, VITO, 2400 Mol, Belgium; 3 Center for Oncological Research, University of Antwerp, 2610 Wilrijk, Belgium; julie.jacobs@uantwerpen.be (J.J.); christophe.deben@uantwerpen.be (C.D.); christophe.hermans@uantwerpen.be (C.H.); glenn.broeckx@uza.be (G.B.); evelien.smits@uza.be (E.S.); patrick.pauwels@uza.be (P.P.); 4 Pathology Department, Antwerp University Hospital, 2650 Edegem, Belgium; 5 Center for Cell Therapy and Regenerative Medicine, Antwerp University Hospital, 2650 Edegem, Belgium; 6 Food & Bio-Based Products, AgResearch Ltd., Lincoln 7674, New Zealand; evelyne.maes@agresearch.co.nz; 7 Thoracic Oncology Department, Antwerp University Hospital, 2650 Edegem, Belgium; jo.raskin@uza.be

**Keywords:** mass spectrometry imaging, immunotherapy, non-small cell lung cancer, predictive protein biomarkers, neutrophil defensins

## Abstract

(1) Background: Therapeutic blocking of the interaction between programmed death-1 (PD-1) with its ligand PD-L1, an immune checkpoint, is a promising approach to restore the antitumor immune response. Improved clinical outcomes have been shown in different human cancers, including non-small cell lung cancer (NSCLC). Unfortunately, still a high number of NSCLC patients are treated with immunotherapy without obtaining any clinical benefit, due to the limitations of PD-L1 protein expression as the currently sole predictive biomarker for clinical use; (2) Methods: In this study, we applied mass spectrometry imaging (MSI) to discover new protein biomarkers, and to assess the possible correlation between candidate biomarkers and a positive immunotherapy response by matrix-assisted laser desorption/ionization (MALDI) MSI in 25 formalin-fixed paraffin-embedded (FFPE) pretreatment tumor biopsies (Biobank@UZA); (3) Results: Using MALDI MSI, we revealed that the addition of neutrophil defensin 1, 2 and 3 as pretreatment biomarkers may more accurately predict the outcome of immunotherapy treatment in NSCLC. These results were verified and confirmed with immunohistochemical analyses. In addition, we provide in-vitro evidence of the immune stimulatory effect of neutrophil defensins towards cancer cells; and (4) Conclusions: With proteomic approaches, we have discovered neutrophil defensins as additional prospective biomarkers for an anti-PD-(L)1 immunotherapy response. Thereby, we also demonstrated that the neutrophil defensins contribute in the activation of the immune response towards cancer cells, which could provide a new lead towards an anticancer therapy.

## 1. Introduction

Advanced non-small cell lung cancer (NSCLC), accounting for 80–85% of all lung cancer cases, is generally linked with a poor prognosis, and is one of the leading causes of cancer-related deaths worldwide for both women and men [1]. Chemotherapy, lately often combined with immunotherapy, is the most frequently used treatment modality in metastatic lung cancer. Targeted therapy, based on genetic tumor alterations, is becoming available for more and more patients, but then fails to cure patients from lung cancer. Immunotherapy has radically changed the landscape of lung cancer treatment, demonstrating durable responses and possibly curation in some patients [2].

To date, most clinical successes have been reached with the blockage of immune checkpoints to restore the antitumor immune response [3]. Immune checkpoints are regulatory cell surface molecules intended to end potentially harmful immune responses, and to prevent autoimmune diseases [4]. One of the most exciting immunotherapies, which is the focus for this study, includes the reactivation of immune T cell-mediated antitumor activity by inhibiting the programmed death-1 (PD-1)/programmed death ligand-1 (PD-L1) interaction [5]. PD-1 is a marker of activated T cells, that interacts with its ligand PD-L1 to downregulate T cell activation, in order to maintain self-tolerance [6]. NSCLC tumor cells can exploit this mechanism and escape immune destruction by expressing PD-L1 on their surface, which through interaction with the regulatory T cell receptor PD-1 causes T cell exhaustion [5,7]. In this regard, the therapeutic blockade of the PD-1/PD-L1 interaction, using agents such as Pembrolizumab [8], Nivolumab [9] or Atezolizumab [10], has shown improved clinical response rates with acceptable adverse events [5,11,12,13]. However, much uncertainty still exists about the unique response patterns of these immunotherapies. Unfortunately, to date, only a subgroup of patients experiences any long term benefit, while severe immune-related toxicities may occur [12,14]. Although different studies suggest that high tumor PD-L1 expression is pivotal for successful cancer immunotherapy [11,15,16], some patients with high PD-L1 expression do not experience benefit from immune checkpoint inhibitors [17], while tumor responses in patients with low or absent tumor PD-L1 expression may be found [18,19]. This clearly demonstrates the limitations of PD-L1 as the sole prospective biomarker currently used in clinical practice [5,20]. There is thus a high need for more effective biomarkers predictive for clinical benefit to anti-PD-(L)1 immunotherapy [17,21,22]. The aim of this study is to establish additional predictive biomarkers for immunotherapy, which will stratify responding NSCLC patients to avoid unnecessary costs and toxicities in patients who will not clinically respond.

We have demonstrated earlier that matrix-assisted laser desorption/ionization mass spectrometry imaging (MALDI MSI) can be a powerful tool to better understand the lung tumor microenvironment on a molecular level [23]. MALDI MSI is a multiplexed analysis that enables the screening of all molecules that can be ionized (i.e., a broad variety of biomolecules, such as peptides, proteins, glycans, nucleic acids, lipids, metabolites, etc.) directly from a single tissue section, without the need for target-specific reagents [24,25,26]. With MALDI MSI, a mass spectrum of each spot on the tissue is generated. This forms a map of the biomolecules (depending on the limit of detection) present in that spot of the tissue. Subsequently, all of the individual recorded mass spectra are merged in one resulting overall average mass spectrum. The measurements are taken in a predefined order, and this allows us to analyze both the distribution and the relative abundances of each biomolecule over the entire tissue section [27]. MALDI MSI produces spatially resolved mass spectrometric data without destroying the tissue morphology, meaning that the same tissue slice can subsequently be hematoxylin and eosin (H&E)-stained, to combine both molecular and histological information [28]. Beside these advantages of MALDI MSI, it is still cumbersome and often difficult to identify interesting MSI targets directly from the tissue itself. In order to overcome this problem, we linked the molecular images with high resolution liquid chromatography (LC)-coupled mass spectrometry, which made it feasible to obtain a reliable identification of interesting peptides/small proteins, while retaining their spatial distribution throughout the lung tissue [23]. Since no prior knowledge of molecular identities is required for MALDI MSI experiments, we used this technology to compare the presence and distribution of naturally-occurring peptides in tumor and peripheral tissue.

By differential expression analysis, we have revealed three interesting antimicrobial peptides, i.e., neutrophil defensin 1, neutrophil defensin 2 and neutrophil defensin 3. These neutrophil defensins belong to the so-called antimicrobial peptides (AMPs) or ‘host defense peptides’ [29,30]. These cytotoxic peptides are produced and released from the granules of neutrophils as a first line of defense to microbial invasions, to fight and eliminate bacterial and/or viral particles, by damaging cell membranes and entering the cells [29]. Neutrophil defensin 1, 2 and 3 were already suggested as potential molecular markers of response to chemotherapy, as defensin overexpression is associated with a response of breast cancer patients to neoadjuvant taxane-based therapies [31]. The most important finding found in literature for our research is the homozygous deletion of the defensin genes in human cancers, which is associated with Ipilimumab (anti-CTLA-4 immunotherapy) resistance [32]. To study the possible association of neutrophil defensin 1, 2 and 3 expression with an anti-PD-(L)1 immunotherapy response, we have applied the newly developed MALDI MSI method to pretreatment biopsies of responding and nonresponding NSCLC patients to anti-PD-(L)1 immunotherapy. From this, it became clear that the expression of these neutrophil defensins was associated with a positive immunotherapy response. Additionally, these proteomic findings were verified with immunohistochemical analyses, which may be used in a clinical setting as a pretreatment biomarker for prediction of the response to immunotherapy [33,34]. Moreover, previous studies have reported that neutrophil defensins act as inducers of tumor necrosis [29,35], and their anticancer activity has been demonstrated in a variety of tumor cells [36]. Although in colorectal cancer, the neutrophil defensins are known markers of development and contribution to colorectal tumor growth [37]. The general association between neutrophil defensins and their role in cancer is still not fully understood. In this study, we provided first insights into the in-vitro biological activity of neutrophil defensin 1, 2 and 3 as immune stimulatory effectors towards lung cancer cells, which may provide a lead towards the underlying biological explanation of the observed prognostic value of neutrophil defensins in lung cancer immunotherapy.

## 2. Results

### 2.1. MALDI Mass Spectrometry Imaging for Predictive Biomarker Discovery

**Figure 1:**
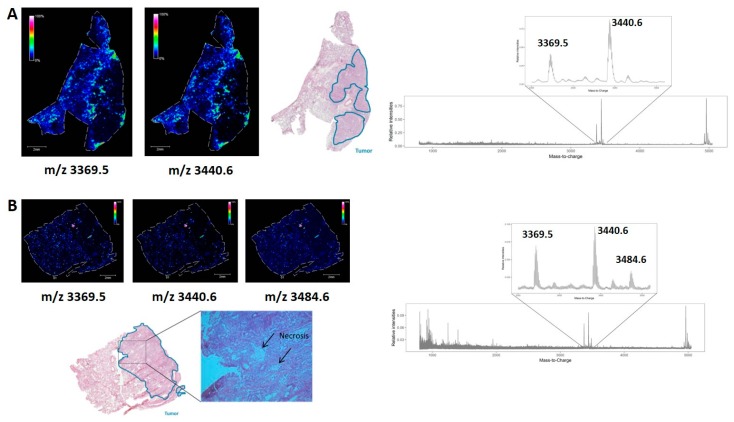
Distribution of the peptides of interest in fresh frozen non-small cell lung cancer (NSCLC) tissues, obtained with matrix-assisted laser desorption/ionization (MALDI) mass spectrometry imaging. (**A**) In this example of human squamous cell carcinoma lung cancerous tissue with adjacent reference tissue, the interesting peptides are expressed in the nontumor region, highly expressed at the interaction border between the nontumor and the tumor region and a low to no expression deeper in the tumor region; (**B**) In this example of human adenocarcinoma lung cancerous tissue with adjacent reference tissue, these peptides are also expressed in necrotic regions within the tumor region. These regions were confirmed in the corresponding hematoxylin and eosin (H&E) staining, and performed after the matrix-assisted laser desorption/ionization mass spectrometry imaging (MALDI MSI) experiment on the same tissue section. The overall average mass spectrum of the whole tissue section obtained after MALDI MSI, which is zoomed out at the peptides of interest *m*/*z* 3369.5, *m*/*z* 3440.6 (and *m*/*z* 3484.6), is displayed, of which the corresponding distribution is portrayed in the left part of the figure.

We have demonstrated earlier that MALDI mass spectrometry imaging (MSI) is a powerful tool to visualize the NSCLC tumor microenvironment based on the endogenous peptidomic profile (*m*/*z* range 800–5000 Da) [23]. The analysis of differential peptide expression in the tumor versus nontumor region revealed two molecules at *m*/*z* 3369.5 and *m*/*z* 3440.6. These show an interesting distribution, with a limited expression in the nontumor region, a high expression at the interaction border between the nontumor and tumor region, and finally a low to no expression deeper in the tumor region, as displayed in Figure 1A, which is an example of a human squamous cell carcinoma fresh frozen lung tissue biopsy. In a fresh frozen lung tissue adenocarcinoma example, a third molecule at *m*/*z* 3484.6 with a similar distribution was observed (Figure 1B), although the expression at the interaction border is not as high as in Figure 1A. Furthermore, three spots of the high expression of these three peptides are observed within the tumor region. Using H&E staining, these regions within the tumor were confirmed as necrotic regions.

### 2.2. Peptides of Interest are Identified as Neutrophil Defensin 1, 2 and 3

Identification of these three peptides of interest was not possible with top-down peptidomics, as performed earlier for an identification of other MALDI MSI targets with higher-energy collisional dissociation (HCD) [23]. Although the precursors for these peptides could be selected for fragmentation, the corresponding fragmentation spectra did not yield sufficient fragments to allow peptide identification. Since electron-transfer dissociation (ETD) has been used successfully in the past for the identification of naturally occurring peptides [38], we applied ETD as a fragmentation technique instead of HCD. The resulting fragmentation spectra of the three target peptides showed a neutral loss of 3 Da in each peptide (presented in Figure S1B). This possibly corresponds to a reduction of three disulfide bridges between two cysteine residues, as it has been proven that ETD can induce disulfide bond cleavage [39,40,41].

To avoid reduction and alkylation steps, which complicates the direct linkage of the *m*/*z* values of intact molecules observed with MALDI MSI, we developed a method where disulfide bridges containing peptides were reduced within a high-resolution mass spectrometer with electron-transfer dissociation (ETD) in an MS^2^ scan (see more details in Figure S1). The resulting reduced intact peptide is then immediately selected for fragmentation with collision-induced dissociation (CID) in the linear trap of the instrument. 

**Figure 2:**
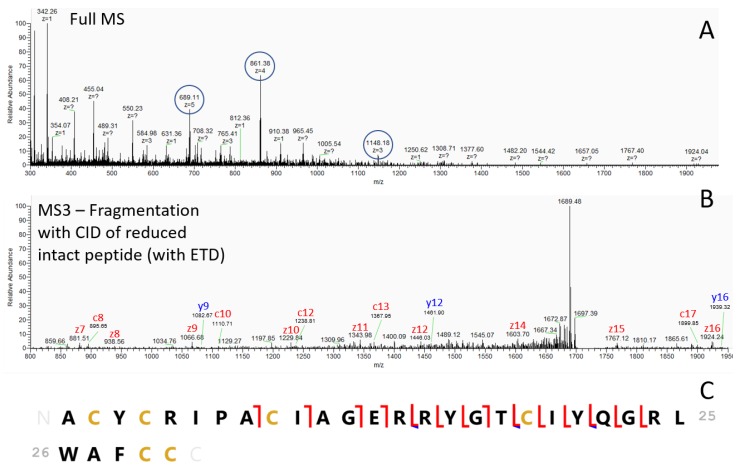
Mass spectra and annotated sequence of neutrophil defensin 1. (**A**) Full mass spectrometry (MS) spectrum of intact neutrophil defensin 1, in three different charge states (3, 4 and 5, indicated by blue circle). The five charged ion with *m*/*z* 688.91 (mass 3439.55 Da as obtained with MSI in Figure 1 is selected for reduction of the three disulfide bridges with electron-transfer dissociation (ETD) and the reduced, intact peptide is immediately selected for fragmentation with collision-induced dissociation (CID); (**B**) The resulting fragmentation spectrum with c, y and z type ions. A sequence tag is depicted on the fragmentation spectrum; (**C**) Annotated sequence of neutrophil defensin 1 with all matched deconvoluted fragments (see also Table 1, mass accuracy was set at 300 ppm) (y ions are indicated in blue, while c and z ions are indicated in red), obtained with both CID as ETD as fragmentation method. See also the Supplementary Materials for more details.

Fragments can be a mixture of b and y ions, obtained by CID fragmentation, and c and z ions, obtained by ETD fragmentation. Manual interpretation of the spectra was performed to obtain sequence tags that allow identification of the full corresponding peptide sequence. Matching the observed fragments with the theoretical fragments of the putative peptide was performed in Prosight lite. Using this approach, we have identified *m*/*z* 3440.6 as neutrophil defensin 1, presented in Figure 2 and Table 1. These findings were confirmed by comparing the same ETD-CID approach with those of synthetic neutrophil defensin 1 (Figure S1 and Table S1). The two other peptides of interest were identified in the same way as neutrophil defensin 2 and 3 (data presented in Tables S2–S5). These three peptides differ from each other in only a single amino acid.

### 2.3. Neutrophil Defensins as Pretreatment Biomarkers for Immunotherapy Response

**Figure 3:**
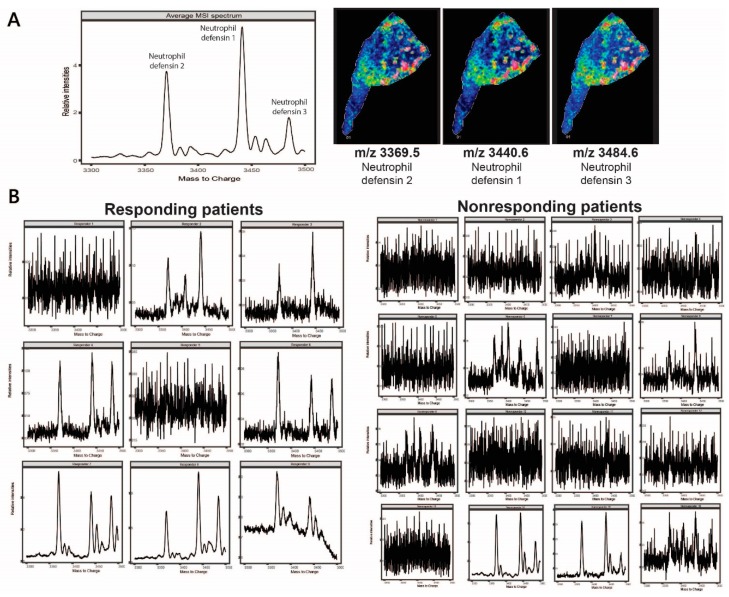
Average MALDI MSI spectra obtained from whole formalin-fixed paraffin-embedded (FFPE) tumor biopsies of NSCLC patients. (**A**) An example of a resulting average mass spectrum with neutrophil defensin 1, 2 and 3 and their corresponding distribution within the tissue, obtained with MALDI MSI analysis on a FFPE tissue section; (**B**) Average MALDI MSI spectra of 25 pretreatment tumor FFPE biopsies from NSCLC patients that received anti-PD-(L)1 immunotherapy. From this small patient cohort, nine patients received clinical benefit from the therapy (responders), from which seven patients showed expression of the neutrophil defensins. The other 16 NSCLC patients did not derive any clinical benefit from immunotherapy treatment (nonresponders), from which 14 show no (or very low) neutrophil defensin expression. From the nonresponding patients, two NSCLC patients showed neutrophil defensin expression.

To evaluate the potential relationship between neutrophil defensin 1, 2 and 3 and the anti-PD-(L)1 immunotherapy response in NSCLC patients, peptidomic profiling was performed on pretreatment formalin-fixed paraffin-embedded (FFPE) biopsies of NSCLC patients who received immunotherapy. So far, MALDI MSI data was limited to fresh frozen lung tissue biopsies in this study (and a previous study [23]), as the visualization of intact immune-related factors is of interest. Due to the formation of inter- and intra-molecular cross-linking of proteins by formalin fixation, analysis of intact peptides and proteins leads to difficulties in FFPE lung tissue biopsies [42]. On the other hand, histological and morphological integrity is preserved in FFPE tissue specimens, which makes these type of tissues suitable for pathological analysis [43]. In addition, FFPE tissue blocks can be stored long-term at room temperature, with only a slight reduction in quality, which is why archives of pretreatment biopsies from large cohorts of patients are available [44]. Remarkably, visualization of the three identified neutrophil defensins was possible with MALDI MSI in FFPE lung tissue biopsies after minimal sample preparation steps (see Materials and Methods). A resulting mass spectrum of the three neutrophil defensins averaged over a tissue slide is depicted in Figure 3A, as well as the distribution of each neutrophil defensin individually. In a next set of experiments, we have assessed the possible correlation between neutrophil defensin expression and a positive immunotherapy response by MALDI MSI in 25 FFPE pretreatment tumor biopsies (Biobank@UZA). These primary tumor biopsies were obtained from NSCLC patients who were treated with immunotherapy (Materials and Methods), based on PD-L1 expression levels. For every patient, the clinical outcome in terms of tumor regression and the acceptable adverse events of immunotherapy was evaluated. Average MALDI MSI profiles of the defensins of each FFPE tumor biopsy is depicted in Figure 3B. In these average MALDI MSI spectra from nine patients responding and 16 patients nonresponding to immunotherapy treatment, an evaluation on the clear presence or absence of ions corresponding to neutrophil defensin 1, neutrophil defensin 2 and neutrophil defensin 3, was performed. Interestingly, the defensin peptides were present in 7 out of 9 pretreatment biopsies of NSCLC patients with a positive response to anti-PD-(L)1 immunotherapy treatment, while the neutrophil defensins were present in only 2 out of 16 nonresponding patients. These results suggest a correlation between neutrophil defensin expression and a positive response to immunotherapy in NSCLC patients. To verify these outcomes in a more quantitative way, immunohistochemistry analyses were performed to allow statistical analysis.

### 2.4. Verification of Biomarkers with Immunohistochemistry Staining (IHC)

**Figure 4:**
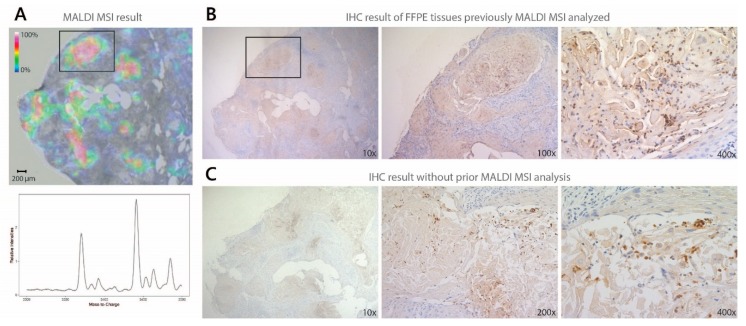
Comparison of defensin 1/3 antibody staining on FFPE tissue sections previously MALDI MSI analyzed and without prior MALDI MSI analysis. (**A**) Distribution of the three neutrophil defensins in FFPE lung tissue obtained with MALDI MSI. The mass spectrum displays the three neutrophil defensins, while the MSI image only displays the distribution of neutrophil defensin 1 (*m*/*z* 3440.6), as neutrophil defensin 1 shows the same distribution as neutrophil defensin 2 (*m*/*z* 3369.5) and 3 (*m*/*z* 3484.6); (**B**) Validation of the presence of the neutrophil defensins obtained with MALDI MSI with immunohistochemical (IHC) analysis. The region indicated with the box in the MSI result was compared with the same tissue region after immunohistochemical analysis with the defensin 1/3 antibody; (**C**) No change in staining intensity is observed compared with the same IHC staining protocol on a consecutive FFPE tissue section with no prior MALDI MSI analysis.

The MALDI MSI results were verified with immunohistochemical (IHC) analyses on the same pretreatment biopsies. IHC analysis of the neutrophil defensins was performed with a defensin 1/3 polyclonal antibody, which cannot make the distinction between the three neutrophil defensins, as opposed to MALDI MSI. Using this latter technique, we can distinguish the three different neutrophil defensins, based upon their difference in mass, even though they only differ in one amino acid. IHC analysis demonstrated the presence of at least one of the neutrophil defensins in positive controls, while no staining of the neutrophil defensins was observed in the neutrophil defensin negative control (Figure S2). In Figure 4, we illustrated that IHC is feasible on FFPE tissue sections, previously analyzed with MALDI MSI, after the matrix was removed with an alcohol wash. In addition, the same IHC protocol was performed on a consecutive FFPE tissue section without prior MSI analysis. Thereof, we may conclude that there is no apparent change in staining intensity compared to IHC performed on tissue sections with and without prior MSI analysis.

**Figure 5:**
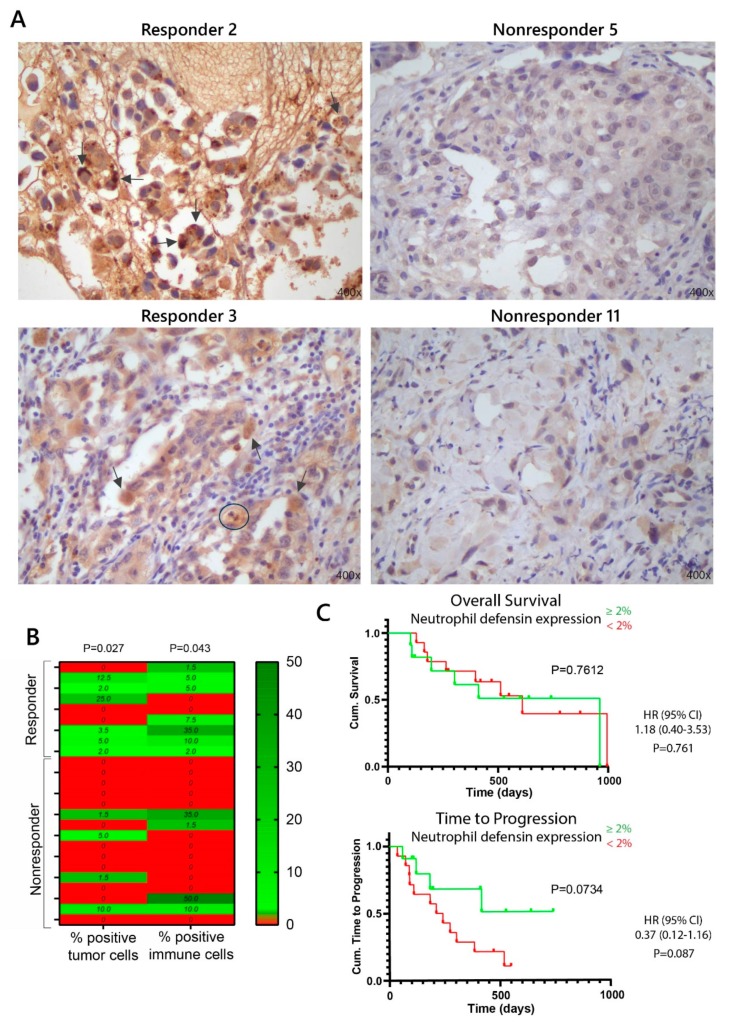
Immunohistochemistry analyses of expression of neutrophil defensin 1/3 on both tumor cells and immune cells in pretreatment tumor biopsies of NSCLC patients who received anti-PD-(L)1 immunotherapy treatment. (**A**) Example of the IHC staining of two different responding patients and two nonresponding patients to immunotherapy treatment. Few examples of neutrophil defensin expression were indicated by the arrows, and an example of expression on neutrophil granulocyte was encircled. Magnification 400×; (**B**) The percentage of neutrophil defensin positive cells for each NSCLC patient individually, determined with IHC. A significant difference between responding and nonresponding patients is observed based upon neutrophil defensin expression on both tumor cells (*p* = 0.027) as on immune cells (*p* = 0.043), examined with a Mann–Whitney U test; (**C**) Kaplan–Meier survival curves for overall survival (OS) and time to progression (TTP) according to neutrophil defensin expression: NSCLC patients were grouped by ≥2% (cut-off 2% determined by receiver operating characteristic (ROC) analysis) neutrophil defensin expression on tumor cells and/or immune cells (green) and <2% neutrophil defensin expression (red). Significance was determined using the log-rank test. Hazard ratio (+95% CI) is determined using Cox regression.

Using this methodology, a pathologist validated each tumor pretreatment biopsy for the presence or absence of the neutrophil defensins on both the tumor cells and immune cells. An example of IHC staining for two responding and two nonresponding NSCLC biopsies is shown in Figure 5A. Although background staining can be observed (as seen in the IHC staining of nonresponding patients in Figure 5A), detection of the neutrophil defensins is feasible, as the staining intensity is clearly higher when neutrophil defensins are present (Figure 4 and Figure 5A for responding patients). The individual neutrophil defensin scoring for both tumor cells and immune cells is shown in Figure 5B. To elucidate whether the neutrophil defensin expression was related to anti-PD-(L)1 immunotherapy response, we performed a Mann–Whitney U test, which showed a significant association between the percentage of neutrophil defensin positive tumor cells and the response to immunotherapy (*p* = 0.027). This association was also observed when the neutrophil defensins were expressed on immune cells (*p* = 0.043), presented in Figure 5B.

We constructed a receiver operating characteristic (ROC) curve for the percentage of neutrophil defensin expression in the immunotherapy responder and nonresponder groups. The area under the curve (AUC) for neutrophil defensin expression on tumor cells and immune cells was 0.740 [95% CI: 0.520–0.959] and 0.736, respectively [95% CI: 0.523–0.949]. The optimal sensitivity and specificity for the detection of responders was obtained with a cut-off of 1.75% (Figure S3). Based on this cut-off value, neutrophil defensin expression was considered positive if there was at least a 2% expression on tumor and immune cells. With this new positivity percentage, χ^2^-tests revealed an even more significant difference between responders and nonresponders based upon tumor cells (*p* = 0.01) and immune cells (*p* = 0.031). These significant differences were highlighted in Table 2. From Table 2, it is clear that neutrophil defensin expression was only associated with response to immunotherapy, not with other clinicopathological parameters. When grouping the patients based on neutrophil defensin expression (≥2% on tumor cells and/or immune cells), univariate survival analysis did not show any difference in overall survival (OS), but a trend towards a longer time before disease progression was shown for NSCLC patients that exhibited neutrophil defensin expression, demonstrated in Figure 5C. Also, neutrophil defensin expression in NSCLC patients may result in a reduced risk to progression of the disease (hazard ratio is 0.37).

### 2.5. Neutrophil Defensins Lead to In Vitro Reduced Lung Tumor Cell Growth

**Figure 6:**
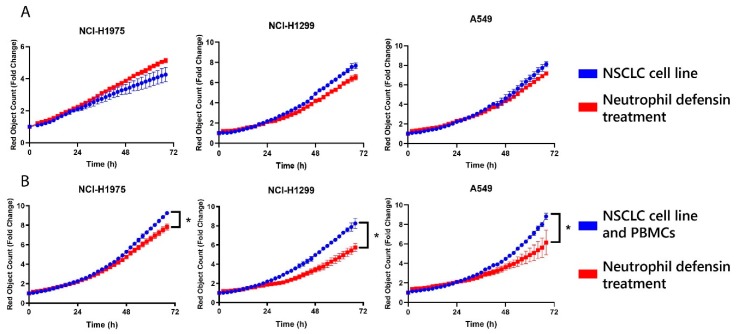
In-vitro analysis of tumor cell survival after addition of neutrophil defensin 1, 2 and 3. Tumor cell survival was determined by follow-up of the red object count using IncuCyte Zoom System. Data is represented as fold change compared to cell count before addition of treatment and the timelapse of the means ± the standard error of the mean (SEM) is displayed for each NSCLC cell line. (**A**) NSCLC cells (NCI-H1975, NCI-H1299, A549) were treated with neutrophil defensin 1, 2 and 3 (Red, 5 µM) or phosphate-buffered saline (PBS) (Blue, control) for 3 days. No concluding results can be made; (**B**) NSCLC cells (NCI-H1975, NCI-H1299, A549) were cocultured with peripheral blood mononuclear cells (PBMC) from three healthy donors and treated with neutrophil defensin 1, 2 and 3 (Red, 5 µM) or PBS (Blue, control) for 3 days. Two replicates for every donor (three donors in total) were measured. Significant decrease (* *p* = 0.05) in tumor cell proliferation for all three NSCLC cell lines.

A possible explanation for the contribution of the neutrophil defensins to the immunotherapy response could be an interplay with adaptive immunity [45]. To confirm this hypothesis, cocultures of NSCLC cell lines (NCI-H1975, NCI-H1299 and A549) and peripheral blood mononuclear cells (PBMC) from healthy donors (effector/target (E/T): 10/1) were treated with either three neutrophil defensins or phosphate-buffered saline (PBS) control. Real-time follow-up was assessed. Treatment with neutrophil defensins significantly decreased tumor cell proliferation in all three NSCLC cell lines (*p* = 0.05, determined after 3 days of treatment), compared to PBMC coculture with PBS control treatment (Figure 6). This effect was most pronounced in NCI-H1299. A conceivable explanation for this observation is immune activation towards cancer cells, as an effect of addition of defensins to the cancer cells was only observed in the presence of PBMC. Direct addition of neutrophil defensins to the three NSCLC cell lines in the absence of immune cells did not result in a significant decrease of tumor cell proliferation, and even an increase in NCI-H1975 cell proliferation was observed (Figure 6A).

**Figure 7:**
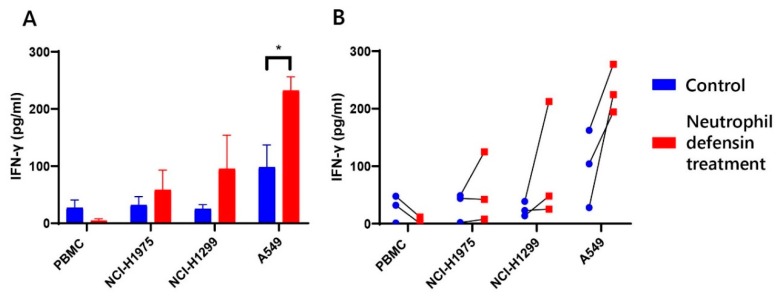
IFN-γ secretion in the supernatants of in-vitro cocultures treated with neutrophil defensin 1, 2 and 3. PBMC alone or cocultured with NSCLC cells (NCI-H1975, NCI-H1299, A549) were treated with neutrophil defensin 1, 2 and 3 (Red, 5 µM) or PBS (Blue, control) for 3 days. Thereafter, supernatants were used to determine the amount of IFN-γ. (**A**) Means ± SEM of PBMC from three healthy donors after 66 h treatment with neutrophil defensins. This results in a significant increase (* *p* = 0.05) of IFN-γ concentration for A549; (**B**) Increase in IFN-γ concentration for each donor individually.

To evaluate the occurrence of immune activation, IFN-γ expression was measured in the supernatants of the PBMC and tumor cocultures treated with neutrophil defensins, as IFN-γ is a key moderator of the activation of cell-mediated immunity [46]. Our results demonstrate a clear increase in IFN-γ secretion after treatment of the coculture with neutrophil defensins (Figure 7). Surprisingly, treatment of neutrophil defensins to PBMC from healthy donors in the absence of NSCLC cells did not result in an increase of IFN-γ release. This seems to indicate that neutrophil defensins contribute to activating the immune response, although not directly. This observation can provide a first lead towards explaining the contribution of the neutrophil defensins to anti-PD-(L)1 immunotherapy response.

## 3. Discussion

Although restoring the antitumor immune response by anti-PD-(L)1 immunotherapy has shown improved clinical outcomes in NSCLC patients, still a high number of NSCLC patients treated with immunotherapy do not respond while being subject to potentially serious toxicity. PD-L1 IHC as a biomarker is only weakly predictive for response to immunotherapy [5,20]. Better predictive markers are urgently needed.

In this study, we used MALDI mass spectrometry imaging to study the distribution of immune-related peptides in relation to the anti-PD-(L)1 immunotherapy response. Since no prior knowledge of molecular identities is required for MALDI MSI experiments, crucial insights regarding immune-related peptide profiles can be obtained directly from tissues. Our differential expression analyses revealed three interesting peptides, namely, neutrophil defensin 1, 2 and 3. These neutrophil defensins 1, 2 and 3, also known as human alpha defensin 1, 2 and 3, or human neutrophil peptide (HNP) 1, 2 and 3, belong to the so-called antimicrobial peptides (AMPs) or ‘host defense peptides’ [29,30]. As mentioned earlier, these cytotoxic peptides are produced and released from the granules of neutrophils as a first line of defense to microbial invasions, to fight and eliminate bacterial and/or viral particles, by damaging cell membranes and entering the cells [29]. More interestingly for this study, neutrophil defensin 1 is associated with the induction of tumor necrosis (see also Figure 1B) when expressed intratumorally, which makes this endogenous peptide a potential prognostic biomarker in cancer [29,35,47]. The three identified neutrophil defensins are also suggested as potential molecular markers of response to chemotherapy, as defensin overexpression is associated with a response of breast cancer patients to neoadjuvant taxane-based therapies [31]. The most important finding according to literature is the homozygous deletion of the defensin genes in human cancers, which is associated with Ipilimumab (anti-CTLA-4 immunotherapy) resistance [32].

Peptidomic profiling with MALDI MSI on NSCLC pretreatment biopsies suggests neutrophil defensin 1, 2 and 3 as potential predictive biomarkers of the response to anti-PD-(L)1 immunotherapy. Immunohistochemical analyses confirmed these findings, and although background staining was relatively high, and needs further optimization, neutrophil defensin staining on single cells can be validated, which cannot be achieved with MALDI MSI (yet). 

We illustrated that IHC is feasible on FFPE tissue sections, previously analyzed with MALDI MSI, which shows the potential to match peptide/protein information without additional consumption of tissue material [34], as pretreatment tissue specimens can be scarce. Our major finding using IHC was the discovery of a significant difference between responders and nonresponders to anti-PD-(L)1 immunotherapy treatment, based upon neutrophil defensin expression on both tumor cells (*p* = 0.027) as immune cells (*p* = 0.043). Further statistical analysis revealed that we can categorize NSCLC patients as a responder when at least 2% of tumor cells (*p* = 0.01) or immune cells (*p* = 0.031) show neutrophil defensin expression. Notwithstanding the limited number of pretreatment biopsy samples available, it offers valuable insights to the classifying power of the three neutrophil defensins as additional predictive biomarkers in anti-PD-(L)1 immunotherapy treatment. With the combined use of PD-L1 and the neutrophil defensins as predictive biomarkers, a much better classification of responding patients could be achieved compared to PD-L1 alone. This is also clear from the longer time to the progression for NSCLC patients who show an expression of neutrophil defensins, demonstrated in the Kaplan–Meier survival curve and by HR of 0.37, meaning a reduced risk to progression of the disease. However, a follow-up study with an increased sample size could further increase the significance of the results. 

Still a few NSCLC patients were wrongly stratified. Responders 1 and 5 would not be stratified as a responding patient based upon neutrophil defensin absence, but nevertheless showed an objective response to immunotherapy. Before they received immunotherapy, they were treated with induction chemotherapy or radiotherapy, possibly resulting in neoantigen release. In addition, both patients were treated with *KRAS* inhibitors, which all resulted in a proinflammatory response. Although, this study has revealed three putative predictive biomarkers that may allow a much better prediction of the immunotherapy response, further research could identify additional predictive biomarkers that may lead to a more complete response pattern, avoiding unnecessary immunotherapy treatment in NSCLC patients.

The importance of neutrophil defensin 1, 2 and 3 was also confirmed with in-vitro physiological data. This study revealed that tumor cell proliferation is significantly reduced in three different NSCLC cell line cocultures with PBMCs in the presence of neutrophil defensins. Furthermore, an increased secretion of the proinflammatory cytokine IFN-γ was observed when the coculture was treated with neutrophil defensin 1, 2 and 3. A possible explanation for this is the activation of the immune response towards cancer cells, as it has been proven earlier that AMPs may activate adaptive immunity [45].

Nevertheless, two limitations of this study may need to be addressed: (1) Only a limited amount of pretreatment biopsies are investigated within this research. At this point, these putative biomarkers were not evaluated with an independent validation set, as a limited amount of pretreatment biopsies with knowledge of response are available due to the only recent introduction of the therapy in the clinic. Although the current study is based on a small sample of biopsies, and future validation is important to confirm these results, the findings suggest the possible association between neutrophil defensin expression and response to anti-PD-(L)1 immunotherapy; (2) The understanding of the underlying cause of the association of the expression of neutrophil defensins and the better response to anti-PD-(L)1 immunotherapy is limited. Although, other studies suggest a direct cytotoxic anticancer activity of the neutrophil defensins [29,35,36], our results suggest a possible immune–stimulatory effect of neutrophil defensin 1, 2 and 3 towards lung cancer. As this is only a proof-of-concept, further research is required to fully understand their mechanism in human cancers, and the eventual use of the neutrophil defensins as a lead in new anticancer therapy, especially as the role of neutrophils, is ambiguous [48,49]. Neutrophil defensins are produced and released by neutrophils [29], as also could be observed in our pretreatment biopsies (indicated by a circle in Figure 5A responder. (3) So far, it has been suggested that neutrophil cells induce a proliferation of NSCLC cells in a dose-dependent manner [48], and tumor-associated neutrophils (TANs) have been associated with poor prognosis [49]. These tumor-promoting functions by neutrophils are mostly investigated in late-stages of tumorigenesis, where chronic inflammation is already developed, but their phenotype and functions seem to vary during tumor progression [50]. Although generally the role of neutrophils in tumors is poorly understood, our findings suggest an important role for neutrophils in tumor immunology. In addition, recent findings indicate the antitumor properties of neutrophils in early-stage human tumors. As they are key players in the inflammatory response, they could improve cytotoxic T cell responses to fight human cancer cells [50].

## 4. Materials and Methods

### 4.1. Study Design

Pretreatment biopsies were collected from 25 NSCLC patients (FFPE tumor tissues), for which the main clinicopathological characteristics are displayed in Table 3.

Currently, only 25 samples were available to meet the data inclusion criteria; these NSCLC patients were selected for treatment with PD-1/PD-L1 antagonists (both Pembrolizumab, Nivolumab and Atezolizumab), based upon ≥50% PD-L1 expression and sufficient tumor-infiltrating lymphocytes (TILs). Nine (36%) had a positive response (i.e., tumor regression determined by response evaluation criteria in solid tumors (RECIST) and acceptable adverse events) to immunotherapy (responders), whereas 16 (64%) had no clinical response (no tumor regression and/or severe toxicities) after treatment (nonresponders). From every NSCLC patient, a primary tumor biopsy is collected, via surgery or lung needle biopsy, to allow comparison in target expression. For each biopsy, two tissue sections were collected on one glass slide for MSI analyses in duplicate, and two consecutive tissue sections were collected for H&E staining and immunohistochemical analysis, respectively. The primary objective was establishing additional predictive biomarkers from NSCLC tissues to better predict the immunotherapy response. The 25 pretreatment biopsies were randomly MSI analyzed, without awareness of the investigator as to whether or not the NSCLC patient had a clinical response to anti-PD-(L)1 immunotherapy. 

Outliers were defined as nonresponders with neutrophil defensin expression or responders without neutrophil defensin expression, and were also included in statistical analyses. From these outliers, medical records were assessed for a possible (medical) explanation. The secondary objective of the biological activity of neutrophil defensins was investigated with in-vitro experiments with three different NSCLC cell lines and PBMC from a buffy coat of healthy donors (approved by the Ethics Committee of the University of Antwerp, reference number 15/48/517), isolated from adult volunteer whole blood donations (supplied by the Red Cross Flanders Blood Service, Belgium). In-vitro experiments were performed at least in duplicate.

The human biological material, both fresh frozen and formalin-fixed paraffin-embedded (FFPE), used in this manuscript was provided by Biobank@UZA (Antwerp, Belgium; ID: BE71030031000); Belgian Virtual Tumorbank funded by the National Cancer Plan [51]. The study was approved by the Ethics Committee of Antwerp University Hospital (15/48/517).

### 4.2. Materials

Acetonitrile, methanol and water (LC-MS graded) were purchased from Biosolve (Valkenswaard, The Netherlands). Ethanol and 2,5-dihydroxybenzoic acid (DHB) were purchased from Merck (Overijse, Belgium). Formic acid, trifluoroacetic acid and Defensin 1/3 polyclonal goat antibody were purchased from Thermo Fisher Scientific (Merelbeke, Belgium). Hexane, xylene and hydrogen peroxide were purchased from Thermo Fisher Acros Organics (Geel, Belgium). The hematoxylin and eosin staining kit, Quick-D mounting medium and formaldehyde were purchased from Klinipath (Olen, Belgium), while the ImmPRESS horseradish peroxidase (HRP) anti-goat antibody polymer detection kit (normal horse blocking serum and secondary anti-goat antibody; Vectorlabs) was obtained from Labconsult (Schaarbeek, Belgium). The 3,3′-Diaminobenzidine (DAB) substrate-chromogen system was purchased from DAKO (Glostrup, Denmark). The synthetic peptides corresponding neutrophil defensin 1, neutrophil defensin 2 and neutrophil defensin 3 were acquired from Synpeptide Co., Ltd. (Shanghai, China). RPMI-1640, Dulbecco’s modified Eagle’s medium (DMEM), fetal bovine serum (FBS), streptomycin and L-glutamin for in-vitro experiments were purchased from Life Technologies (Merelbeke, Belgium). A V-PLEX proinflammatory IFN-γ (human) kit was purchased from Meso Scale Discovery (Rockville, MD, USA). Non-small cell lung cancer cell lines NCI-H1975, NCI-H1299 and A549 were purchased from the American type cell culture collection (ATCC, Manassas, VA, USA). The MYCOALERT^®^ Mycoplasma detection kit was obtained from Lonza (Basel, Suisse) and Nuclight red from Essen Bioscience (Welwyn Garden City, Hertfordshire, UK).

### 4.3. Fresh Frozen Tissue Sectioning and Preparation for MSI Analysis

Fresh frozen human lung cancerous tissue sectioning was performed on a LEICA CM1950UV cryostat to obtain sections of 14 µm thickness, and were thaw-mounted on Indium Tin Oxide (ITO)-coated glass slides (Bruker Daltonik GmBH, Bremen, Germany). Tissue sections were treated according to Carnoy’s washing procedure (30 s in 70% EtOH, 30 s in 100% EtOH, 90 s in Carnoy’s fluid (EtOH:acetic acid:water (90:9:1 *v*:*v*:*v*)) and 30 s in 100% EtOH), dried for 30 min and 12 layers of 2,5-dihydroxybenzoic acid (DHB) matrix (40 mg/mL in 60/0.1 (*v*/*v*) acetonitrile/trifluoroacetic acid) was deposited on the tissue by using a SunCollect pneumatic sprayer (SunChrom, Friedrichsdorf, Germany). MALDI MSI data were acquired with a rapifleX tissue typer in single time of flight (TOF) mode (Bruker Daltonik GmBH, Bremen, Germany), as described earlier [23]. The resulting mass spectra will be further processed in R software (Cardinal) [52].

### 4.4. AMP Identification

In order to identify interesting MSI targets, small lung tissue blocks were homogenized, and peptides were extracted and dissolved, as described before [23]. After separation by reversed phase C18 (RP-C18) liquid chromatography on a nanoAcquity UPLC system (Waters, Milford, MA, USA), the interesting three MSI targets were identified on a LTQ Velos Orbitrap mass spectrometer equipped with a nanospray Flex Ion source (Thermo Fisher, Waltham, MA, USA). The high-resolution mass spectrometer was set up in data-dependent acquisition mode, with an automatic gain control (AGC) target of 1 × 10^5^, and the maximum injection was set to 500 ms. The precursor ion for the ETD scan was isolated in data-dependent acquisition mode with an isolation window width of 3. Activation time for the reduction of this isolated ion with ETD was 200 ms, and the reduced ion was selected (isolation window of 4) for further CID fragmentation in the CID MS^3^ step with a normalized collision energy of 45% in CID (activation time of 70 ms). Fragmentation spectra of the selected ions were displayed in Xcalibur (Thermo Fisher, Waltham, MA, USA), and fragment masses and corresponding intensities were exported as CSV files for manual de novo sequencing, aided and annotated by Prosight lite [53]. Settings were 300 ppm mass accuracy for the ion trap mode. Sequences were obtained from the Uniprot human database.

### 4.5. FFPE Tissue Sectioning and Preparation for MSI Analysis

Formalin-fixed paraffin-embedded (FFPE) human lung cancerous tissues were collected, and tissue sectioning was performed on a HM 340E (Microm) (Thermo Fisher Scientific, Merelbeke, Belgium) to obtain sections of 5 µm thickness. The paraffin ribbon was placed in a dH_2_O bath at room temperature. Individual tissue sections were straightened in a water bath of 50 ± 2 °C, and mounted on Indium Tin Oxide (ITO)-coated glass slides (Bruker Daltonik GmBH, Bremen, Germany), then allowed to dry on a warming surface of 37 °C for at least 8 h [54]. For each patient sample, two tissue sections were collected on one ITO-coated glass slide. To visualize the three neutrophil defensins in FFPE tissue sections with MALDI MSI, tissue sections were deparaffinized by immersing twice in xylene for each 5 min. Rehydration of the tissue sections was performed by immersing in graded ethanol series (twice immersing in 100% (*vol*/*vol*) ethanol, once 95% (*vol*/*vol*) ethanol and one time 70% (*vol*/*vol*) ethanol, each step for 1 min). The last rinsing step was a 3 min washing step in Milli-Q-purified water, performed twice. Glass slides were air-dried for 30 min. Matrix deposition was the same as described above for fresh frozen tissue sections. MALDI MSI data were acquired with a rapifleX tissue typer in single TOF mode (Bruker Daltonik GmBH, Bremen, Germany), equipped with a SmartBeam 3D laser. Mass spectra were the sum of 1000 individual laser shots, with a 95% laser intensity. Mass spectral neutrophil defensin (*m*/*z* range 2400–5800 Da) images were obtained in positive linear mode with a linear voltage of 3570 V, a sample rate of 0,63 GS/s, a laser resolution of 100 µm and a raster width of 100 µm × 100 µm. All of the spectra are preprocessed with a Top Hat baseline algorithm for baseline subtraction, and the resulting overall average spectrum of the ion image is TIC-normalized in FlexImaging 5.0 (Bruker Daltonik GmBH, Bremen, Germany), and was further processed in R software (Cardinal) [52].

### 4.6. H&E Staining

For every tissue biopsy, one section was hematoxylin and eosin (H&E)-stained according to conventional protocols [39]. For previously MSI analyzed tissue sections, the matrix was removed with 70% (*vol*/*vol*) ethanol, after which the tissue was dried using a vacuum desiccator, and H&E-stained according to conventional protocols. The tissue sections were in this way re-evaluated by the same pathologist of Antwerp University Hospital (UZA) for confirmation of the identity of the observed regions.

### 4.7. Immunohistochemistry of Neutrophil Defensins

Immunohistochemistry of the neutrophil defensins was performed with Defensin 1/3 Polyclonal Goat Antibody. Serial lung FFPE specimens, not previously analyzed by MALDI MSI, were first incubated for 2 h at 60 °C, and subsequently deparaffinized in xylene, rehydrated in serial ethanol solutions and water. Matrix layers of tissue sections after MSI analyses were removed by 70% (*vol*/*vol*) ethanol. Antigen unmasking of all tissue sections was performed by heat-induced epitope retrieval (HIER) by incubation in citrate buffer (pH 6) at 96 °C for 20 min. 

Endogenous peroxidase activity is inactivated by the treatment of 3.5% hydrogen peroxidase for 10 min, and non-specific binding was blocked with 2.5% normal horse blocking serum for 20 min. Incubation of primary antibody at a final 1/100 dilution lasted 1h at room temperature in a humidified chamber, followed by an incubation of 30 min with HRP-conjugated anti-goat secondary antibody. Samples were incubated with DAB substrate chromogen for 5 minutes and counterstained with hematoxylin, dehydrated and mounted. Both positive and negative controls were included (Figure S2). Scoring was performed by a pathologist: Due to weak background staining, the threshold of positive staining was set at moderate staining, while edges of the NSCLC tissue were ignored. Positive staining was assigned when at least 1% of either the tumor cells or immune cells showed specific neutrophil defensin 1/3 staining. Association between the immunotherapy outcome and neutrophil defensin expression on tumor cells and/or immune cells was assessed using the Mann–Whitney U test. χ^2^-analyses were used to determine the statistical significance of neutrophil defensin positivity (cut-off 2% determined by ROC analysis) on response to immunotherapy. All analyses were performed using SPSS version 26 (SPSS Inc., Brussels, Belgium), and significance was reached if *p* ≤ 0.05. Prognostic value of the presence of neutrophil defensins (≥2% on tumor cells or immune cells, as determined by ROC analysis) was assessed by univariate survival analysis, performed in GraphPad using the Kaplan–Meier method. Time to progression (TTP) was defined as cancer progression as an end-point (not including deaths) and overall survival (OS) as the time until death occurred. Statistical significance was determined using the log-rank test. Hazard ratio and its 95% confidence interval (CI) of the defensins as biomarker was fitted using proportional hazards regression (Cox regression). 

### 4.8. Tumor Cell Cultures, Coculturing and Cytokine (IFN-γ) Detection

Non-small cell cancer cell lines NCI-H1975 and NCI-H1299 were cultured in RPMI-1640 media (supplemented with 10% fetal bovine serum (FBS), 1% penicillin/streptomycin and 1% L-glutamine). A549 cells were cultured in DMEM supplemented as described above. Cells were grown as monolayers and maintained in a 5% CO_2_/95% air humidified environment at 37 °C. Cell cultures were regularly tested for the absence of mycoplasma using the MYCOALERT^®^ Mycoplasma detection kit. To obtain red fluorescent nuclei, all NSCLC cell lines were lentivirally transduced using the IncuCyte Nuclight Red lentivirus reagent.

Human PBMCs, provided by the blood transfusion center of the Red Cross (Flanders), were isolated by Ficoll-Paque Plus gradient. In all experiments, PBMCs of three different healthy donors were used.

To evaluate the effect of neutrophil defensin 1, 2 and 3 on immune activation, NCI-H1975 tumor cell lines were seeded at 2500 cells/well, NCI-H1299 at 1500 cells/well and A549 at 2000 cells/well in 96 well plates (Corning, BD). After overnight incubation, co-culture experiments with PBMCs (E/T 10:1) were set-up and treated with 5 µM neutrophil defensin 1, 2 and 3, dissolved in PBS, or with PBS for 3 days. Tumor cell growth, measured by the red object count, was followed every 2 hours with the IncuCyte Zoom System (Essen Bioscience, Welwyn Garden City, Hertfordshire, UK). For all experiments, two replicates of the same condition (for every donor, three donors in total) were measured.

For interferon gamma (IFN-γ) detection, 80,000 cells/well of every tumor cell line was cultured in a 6-well plate for 24 h. Co-cultures with PBMC (E/T 20:1) were set-up and treated with 5 µM neutrophil defensin 1, 2 and 3 or PBS for 3 days, after which the supernatants was collected. Detection of IFN-γ was performed with V-PLEX proinflammatory IFN-γ (human) kit according to the manufacturer’s protocol (Meso Scale Discovery (MSD, Rockville, MD, USA).

All data is presented as the means ± SEM, performed in GraphPad Prism 8 software and significant differences (*p* ≤ 0.05) were analyzed using the Mann–Whitney U test, performed in SPSS version 26 (SPSS Inc., Brussels, Belgium).

## 5. Conclusions

This study is another example of using proteomic approaches for the discovery of new biomarkers and possible new cancer therapeutics, with the advantages that no prior knowledge is required, and the detection of numerous molecules is possible directly from the tissue itself [55,56,57]. Due to the high speed of analyses, ease of use and relatively high sensitivity, MALDI MSI is suitable as a high-throughput screening method, which can be implemented in clinical and pathological settings for therapy decision to allow rapid and multiplexed biomarker screening. The main goal of the current study was to determine additional predictive biomarkers for immunotherapy response with MALDI MSI. Besides the discovery of neutrophil defensin 1, 2 and 3 as additional biomarkers for a better classification of responding patients, we also demonstrated that neutrophil defensin 1, 2 and 3 contribute in activation of the immune response towards cancer cells, which could provide a new lead towards an anticancer therapy.

## 6. Patents

The presented predictive biomarker data was submitted for patenting (EP 19197602.6).

## Figures and Tables

**Table 1:** List of annotated fragment ions in the deconvoluted fragmentation spectrum. The mixture of c, y and z fragment ions were aligned to the amino acid sequence of neutrophil defensin 1, generated with Prosight lite with 300 ppm mass accuracy.

Neutrophil Defensin 1 ACYCRIPACIAGERRYGTCIYQGRLWAFCC *m*/*z* 688.91; *z* = 5; Mass 3439.55 Da Retention Time 31.38 min						
Name	Ion Type	Ion Number	Theoretical Mass	Observed Mass	Mass Difference (Da)	Mass Difference (ppm)
c8	c	8	894.42016	894.580505	0.160345	179.2725692
c10	c	10	1110.51341	1110.714966	0.201556	181.4980334
c12	c	12	1238.57198	1238.811157	0.239177	193.1070651
c13	c	13	1367.61457	1367.952881	0.338311	247.3730592
c17	c	17	1899.90158	1899.846436	−0.055144	−29.02466137
y9	y	9	1082.478985	1082.668701	0.189716	175.2606772
y12	y	12	1461.635565	1461.895508	0.259943	177.843921
y16	y	16	1938.869145	1939.321045	0.4519	233.0740067
z7	z	7	881.38018	881.510986	0.130806	148.4104169
z8	z	8	938.40164	938.560364	0.158724	169.1429269
z9	z	9	1066.46022	1066.680298	0.220078	206.3630653
z10	z	10	1229.52355	1229.841064	0.317514	258.2414953
z11	z	11	1342.60761	1342.919067	0.311457	231.9791707
z12	z	12	1445.6168	1446.032227	0.415427	287.3700693
z14	z	14	1603.68594	1603.703613	0.017673	11.02023754
z15	z	15	1766.74927	1767.123413	0.374143	211.769155
z16	z	16	1922.85038	1923.075928	0.225548	117.2987781

**Table 2:** Association of neutrophil defensin 1/3 expression with clinicopathological characteristics.

	Neutrophil Defensin Expression on Tumor Cells			Neutrophil Defensin Expression on Immune Cells		
	Low (<2%)	High (≥2%)	*p*-Value	Low (<2%)	High (≥2%)	*p*-Value
Gender						
Female	8 (47.1%)	3 (37.5%)	1	9 (56.3%)	2 (22.2%)	0.21
Male	9 (52.9%)	5 (62.5%)		7 (43.7%)	7 (77.8%)	
Histologic type						
Adenocarcinoma	13 (76.5%)	4 (50%)	0.22	12 (75%)	5 (55.6%)	0.33
Squamous	4 (23.5%)	3 (37.5%)		3 (18.7%)	4 (44.4%)	
Large cell		1 (12.5%)		1 (6.3%)		
Stage						
IIb	1 (5.9%)		0.15		1 (11.1%)	0.09
IIIa		2 (25%)			2 (22.2%)	
IVa	11 (64.7%)	5 (62.5%)		11 (68.8%)	5 (55.6%)	
IVb	5 (29.4%)	1 (12.5%)		5 (31.2%)	1 (11.1%)	
Smoking status						
Never-smoker	2 (11.8%)	1 (12.5%)	1	1 (6.25%)	2 (22.2%)	0.53
Smoker	14 (82.3%)	7 (87.5%)		14 (87.5%)	7 (77.8%)	
Unknown	1 (5.9%)			1 (6.25%)		
Therapy response						
Responder	3 (17.6%)	6 (75%)	0.01	3 (18.7%)	6 (66.7%)	0.031
Non-responder	14 (82.4%)	2 (25%)		13 (81.3%)	3 (33.3%)	

**Table 3:** Overview of clinico-pathological characteristics of NSCLC patients who received immunotherapy treatment.

	Responding NSCLC Patients *n* = 9 (%)	Nonresponding NSCLC Patients *n* = 16 (%)
Gender		
Female	4 (44%)	7 (44%)
Male	5 (56%)	9 (56%)
Histologic type		
Adenocarcinoma	6 (67%)	11 (69%)
Squamous	2 (22%)	5 (31%)
Large cell	1 (11%)	
Stage		
IIb		1 (6%)
IIIa	2 (22%)	
IVa	5 (56%)	11 (69%)
IVb	2 (22%)	4 (25%)
Smoking status		
Never-smoker	2 (22%)	1 (6%)
Smoker	7 (78%)	14 (88%)
Unknown		1 (6%)
Genetic aberrations		
KRAS	1 (11%)	2 (13%)
BRAF	1 (11%)	
EGFR		2 (13%)
Immunotherapy		
Pembrolizumab	7 (78%)	7 (44%)
Nivolumab	1 (11%)	8 (50%)
Atezolizumab	1 (11%)	1 (6%)
Median survival		
1-year OS rate	6 (67%)	9 (56%)
1-year PFS rate	6 (67%)	3 (19%)

## Supplementary Materials

The following are available online at https://www.mdpi.com/2072-6694/12/4/863/s1, Figure S1: Mass spectra and annotated sequence of synthetic peptide corresponding for neutrophil defensin 1. (A) Full MS spectrum of intact neutrophil defensin 1, in three different charge states. The fifth charged ion with *m*/*z* 689.31 (mass 3,441.6 Da) is selected for reduction of the three internal disulfide bridges with ETD (activation time 200 ms); (B) The resulting intact peptide *m*/*z* 1722.77 has lost three charges, one for reducing each disulfide bridge. This reduced peptide is immediately selected for fragmentation with CID (activation energy 45 for 70 ms); (C) The resulting fragmentation spectrum with b, c, y and z type ions. The main important ions are depicted on the fragmentation spectrum to confirm the identification of neutrophil defensin 1 (same ions, except for y9, y16 and c17, are detected in Figure 2B); (D) Annotated sequence of neutrophil defensin 1 with all matched deconvoluted fragments (see also Table S1) (b and y ions are indicated in blue, while c and z ions are indicated in red), obtained with both CID and ETD as fragmentation method. Figure S2: Examples of immunohistochemistry (IHC) results for defensin 1/3 antibody staining. (A) Example of defensin 1/3 IHC negative case; (B) Example of defensin 1/3 IHC positive case. Magnification 400×. Figure S3: Receiver operating characteristic (ROC) curve for neutrophil defensin expression on tumor cells and immune cells within responders and nonresponders to immunotherapy. (A) ROC curve and corresponding Area under the Curve (AUC); (B) Sensitivity and specificity for the detection of responders. The optimal was obtained with a cut-off of 1.75% for both cells, indicated in red. Table S1: List of annotated fragment ions in the deconvoluted fragmentation spectrum of the synthetic peptide corresponding neutrophil defensin 1. The mixture of b, c, y and z fragment ions were aligned to the amino acid sequence of neutrophil defensin 1, generated with Prosight lite with 300 ppm mass accuracy. Table S2: List of annotated fragment ions in the deconvoluted fragmentation spectrum. The mixture of c, y and z fragment ions were aligned to the amino acid sequence of neutrophil defensin 2, generated with Prosight lite with 300 ppm mass accuracy. Table S3: List of annotated fragment ions in the deconvoluted fragmentation spectrum of the synthetic peptide corresponding neutrophil defensin 2. The mixture of b, c, y and z fragment ions were aligned to the amino acid sequence of neutrophil defensin 2, generated with Prosight lite with 300 ppm mass accuracy. Table S4: List of annotated fragment ions in the deconvoluted fragmentation spectrum. The mixture of b, c, y and z fragment ions were aligned to the amino acid sequence of neutrophil defensin 3, generated with Prosight lite with 300 ppm mass accuracy. Table S5: List of annotated fragment ions in the deconvoluted fragmentation spectrum of the synthetic peptide corresponding neutrophil defensin 3. The mixture of b, c, y and z fragment ions were aligned to the amino acid sequence of neutrophil defensin 3, generated with Prosight lite with 300 ppm mass accuracy.

## References

[1] International Agency for Research on Cancer Globocan. http://Gco.Iarc.Fr/Today.

[2] Garon E.B., Hellmann M.D., Rizvi N.A., Carcereny E., Leighl N.B., Ahn M.J., Eder J.P., Balmanoukian A.S., Aggarwal C., Horn L. (2019). Five-year overall survival for patients with advanced non-small-cell lung cancer treated with pembrolizumab: Results from the phase i KEYNOTE-001 study. J. Clin. Oncol..

[3] Pardoll D.M. (2012). The blockade of immune checkpoints in cancer immunotherapy. Nat. Rev. Cancer.

[4] Johnson D.B., Peng C., Sosman J.A. (2015). Nivolumab in melanoma: Latest evidence and clinical potential. Ther. Adv. Med. Oncol..

[5] Teixidó C., Vilariño N., Reyes R., Reguart N. (2018). PD-L1 expression testing in non-small cell lung cancer. Ther. Adv. Med. Oncol..

[6] Riley J.L. (2009). PD-1 signaling in primary T cells. Immunol. Rev..

[7] Mellman I., Coukos G., Dranoff G. (2014). Cancer immunotherapy comes of age. Nature.

[8] U.S. Food & Drug Administration (FDA) https://www.fda.gov/Drugs/InformationOnDrugs/ApprovedDrugs/ucm617471.htm.

[9] U.S. Food & Drug Administration (FDA) https://www.fda.gov/Drugs/InformationOnDrugs/ApprovedDrugs/ucm617370.htm.

[10] U.S. Food & Drug Administration (FDA) https://www.fda.gov/drugs/informationondrugs/approveddrugs/ucm525780.htm.

[11] Garon E.B., Rizvi N.A., Hui R., Leighl N., Balmanoukian A.S., Eder J.P., Patnaik A., Aggarwal C., Gubens M., Horn L. (2015). Pembrolizumab for the treatment of non–small-cell lung cancer. N. Engl. J. Med..

[12] Davies M. (2014). New modalities of cancer treatment for NSCLC: Focus on immunotherapy. Cancer Manag. Res..

[13] Blair H.A. (2018). Atezolizumab: A review in previously treated advanced non-small cell lung cancer. Target. Oncol..

[14] Tray N., Weber J.S., Adams S. (2018). Predictive biomarkers for checkpoint immunotherapy: Current status and challenges for clinical application. Cancer Immunol. Res..

[15] Khunger M., Hernandez A.V., Pasupuleti V., Rakshit S., Pennell N.A., Stevenson J., Mukhopadhyay S., Schalper K., Velcheti V. (2017). Programmed cell death 1 (PD-1) ligand (PD-L1) expression in solid tumors as a predictive biomarker of benefit from PD-1/PD-L1 axis inhibitors: A systematic review and meta-analysis. JCO Precis. Oncol..

[16] Velcheti V., Schalper K.A., Carvajal D.E., Rimm D.L., Anagnostou V.K., Syrigos K.N., Sznol M., Herbst R.S., Gettinger S.N., Chen L. (2014). Programmed death ligand-1 expression in non-small cell lung cancer. Lab. Investig..

[17] Taube J.M., Klein A., Brahmer J.R., Xu H., Pan X., Kim J.H., Chen L., Pardoll D.M., Topalian S.L., Anders R.A. (2015). Association of PD-1, PD-1 ligands, and other features of the tumor immune microenvironment with response to anti-PD-1 therapy. Clin. Cancer Res..

[18] Robert C., Long G.V., Brady B., Dutriaux C., Maio M., Mortier L., Hassel J.C., Rutkowski P., McNeil C., Kalinka-Warzocha E. (2015). Nivolumab in previously untreated melanoma without BRAF mutation. N. Engl. J. Med..

[19] Daud A.I., Wolchok J.D., Robert C., Hwu W.J., Weber J.S., Ribas A., Hodi F.S., Joshua A.M., Kefford R., Hersey P. (2016). Programmed death-ligand 1 expression and response to the anti-programmed death 1 antibody pembrolizumab in melanoma. J. Clin. Oncol..

[20] Vilain R.E., Menzies A.M., Wilmott J.S., Kakavand H., Madore J., Guminski A., Liniker E., Kong B.Y., Cooper A.J., Howle J.R. (2017). Dynamic changes in PD-L1 expression and immune infiltrates early during treatment predict response to PD-1 blockade in Melanoma. Clin. Cancer Res..

[21] Jacobs J., Zwaenepoel K., Rolfo C., Van den Bossche J., Deben C., Silence K., Hermans C., Smits E., Van Schil P., Lardon F. (2015). Unlocking the potential of CD70 as a novel immunotherapeutic target for non-small cell lung cancer. Oncotarget.

[22] Teixidó C., Karachaliou N., González-Cao M., Morales-Espinosa D., Rosell R. (2015). Assays for predicting and monitoring responses to lung cancer immunotherapy. Cancer Biol. Med..

[23] Berghmans E., Van Raemdonck G., Schildermans K., Willems H., Boonen K., Maes E., Mertens I., Pauwels P., Baggerman G. (2019). MALDI mass spectrometry imaging linked with top-down proteomics as a tool to study the non-small-cell lung cancer tumor microenvironment. Methods Protoc..

[24] Chughtai K., Heeren R.M.A. (2011). Mass spectrometric imaging for biomedical tissue analysis—Chemical reviews (ACS Publications). Chem. Rev..

[25] Ahlf Wheatcraft D.R., Liu X., Hummon A.B. (2014). Sample preparation strategies for mass spectrometry imaging of 3D cell culture models. J. Vis. Exp..

[26] Minerva L., Clerens S., Baggerman G., Arckens L. (2008). Direct profiling and identification of peptide expression differences in the pancreas of control and ob/ob mice by imaging mass spectrometry. Proteomics.

[27] Stoeckli M., Chaurand P., Hallahan D.E., Caprioli R.M. (2001). Imaging mass spectrometry: A new technology for the analysis of protein expression in mammalian tissues. Nat. Med..

[28] Minerva L., Boonen K., Menschaert G., Landuyt B., Baggerman G., Arckens L. (2011). Linking mass spectrometric imaging and traditional peptidomics: A validation in the obese mouse model. Anal. Chem..

[29] Gaspar D., Freire J.M., Pacheco T.R., Barata J.T., Castanho M.A.R.B. (2015). Apoptotic human neutrophil peptide-1 anti-tumor activity revealed by cellular biomechanics. Biochim. Biophys. Acta-Mol. Cell Res..

[30] Xu N., Wang Y.-S., Pan W.-B., Xiao B., Wen Y.-J., Chen X.-C., Chen L.-J., Deng H.-X., You J., Kan B. (2008). Human -defensin-1 inhibits growth of human lung adenocarcinoma xenograft in nude mice. Mol. Cancer Ther..

[31] Bauer J.A., Chakravarthy A.B., Rosenbluth J.M., Mi D., Seeley E.H., Granja-Ingram N.D.M., Olivares M.G., Kelley M.C., Mayer I.A., Meszoely I.M. (2010). Identification of markers of taxane sensitivity using proteomic and genomic analyses of breast tumors from patients receiving neoadjuvant paclitaxel and radiation. Clin. Cancer Res..

[32] Ye Z., Dong H., Li Y., Ma T., Huang H., Leong H.-S., Eckel-Passow J., Kocher J.-P.A., Liang H., Wang L. (2018). Prevalent homozygous deletions of type I interferon and defensin genes in human cancers associate with immunotherapy resistance. Clin. Cancer Res..

[33] Mukae H., Iiboshi H., Nakazato M., Hiratsuka T., Tokojima M., Abe K., Ashitani J., Kadota J., Matsukura S., Kohno S. (2002). Raised plasma concentrations of α-defensins in patients with idiopathic pulmonary fibrosis. Thorax.

[34] Stenzinger A., Schwamborn K., Kazdal D., Fresnais M., Schirmacher P., Casadonte R., Leichsenring J., Kriegsmann M., Kriegsmann K., Zgorzelski C. (2018). Combined immunohistochemistry after mass spectrometry imaging for superior spatial information. PROTEOMICS–Clin. Appl..

[35] Müller C.A., Markovic-Lipkovski J., Klatt T., Gamper J., Schwarz G., Beck H., Deeg M., Kalbacher H., Widmann S., Wessels J.T. (2002). Human α-defensins HNPs-1, -2, and -3 in renal cell carcinoma. Am. J. Pathol..

[36] Ferdowsi S., Pourfathollah A.A., Amiri F., Rafiee M.H., Aghaei A. (2019). Evaluation of anticancer activity of α-defensins purified from neutrophils trapped in leukoreduction filters. Life Sci..

[37] Mothes H., Melle C., Ernst G., Kaufmann R., Von Eggeling F., Settmacher U. (2008). Human neutrophil peptides 1-3—Early markers in development of colorectal adenomas and carcinomas. Dis. Markers.

[38] Sasaki K., Osaki T., Minamino N. (2013). Large-scale identification of endogenous secretory peptides using electron transfer dissociation mass spectrometry. Mol. Cell. Proteom..

[39] Cole S.R., Ma X., Zhang X., Xia Y. (2012). Electron transfer dissociation (ETD) of peptides containing intrachain disulfide bonds. J. Am. Soc. Mass Spectrom..

[40] Wu S.-L., Jiang H., Lu Q., Dai S., Hancock W.S., Karger B.L. (2009). Mass spectrometric determination of disulfide linkages in recombinant therapeutic proteins using on-line LC-MS with electron transfer dissociation (ETD). Anal. Chem..

[41] Compton P.D., Strukl J.V., Bai D.L., Shabanowitz J., Hunt D.F. (2012). Optimization of electron transfer dissociation via informed selection of reagents and operating parameters. Anal. Chem..

[42] Metz B., Kersten G.F.A., Baart G.J.E., De Jong A., Meiring H., Ten Hove J., Van Steenbergen M.J., Hennink W.E., Crommelin D.J.A., Jiskoot W. (2006). Identification of formaldehyde-induced modifications in proteins: Reactions with insulin. Bioconjug. Chem..

[43] Rahimi F., Shepherd C.E., Halliday G.M., Geczy C.L., Raftery M.J. (2006). Antigen-epitope retrieval to facilitate proteomic analysis of formalin-fixed archival brain tissue. Anal. Chem..

[44] Föll M.C., Fahrner M., Oria V.O., Kühs M., Biniossek M.L., Werner M., Bronsert P., Schilling O. (2018). Reproducible proteomics sample preparation for single FFPE tissue slices using acid-labile surfactant and direct trypsinization. Clin. Proteom..

[45] Ganz T. (2003). The role of antimicrobial peptides in innate immunity. Integr. Comp. Biol..

[46] Bhat P., Leggatt G., Waterhouse N., Frazer I.H. (2017). Interferon-γ derived from cytotoxic lymphocytes directly enhances their motility and cytotoxicity. Cell Death Dis..

[47] Bateman A., Singh A., Jothy S., Fraser R., Esch F., Solomon S. (1992). The levels and biologic action of the human neutrophil granule peptide HP-1 in lung tumors. Peptides.

[48] Hattar K., Franz K., Ludwig M., Sibelius U., Wilhelm J., Lohmeyer J., Savai R., Subtil F.S.B., Dahlem G., Eul B. (2014). Interactions between neutrophils and non-small cell lung cancer cells: Enhancement of tumor proliferation and inflammatory mediator synthesis. Cancer Immunol. Immunother..

[49] Kim Y., Lee D., Lee J., Lee S., Lawler S. (2019). Role of tumor-associated neutrophils in regulation of tumor growth in lung cancer development: A mathematical model. PLoS ONE.

[50] Lecot P., Sarabi M., Pereira Abrantes M., Mussard J., Koenderman L., Caux C., Bendriss-Vermare N., Michallet M.-C. (2019). Neutrophil heterogeneity in cancer: From biology to therapies. Front. Immunol..

[51] BE71030031000 Biobank@UZA, Belgian Virtual Tumourbank funded by the National Cancer Plan. https://virtualtumourbank.kankerregister.org/tumourbank.aspx?url=BVT_home.

[52] Bemis K.D., Harry A., Eberlin L.S., Ferreira C., Van De Ven S.M., Mallick P., Stolowitz M., Vitek O. (2015). Cardinal: An R package for statistical analysis of mass spectrometry-based imaging experiments. Bioinformatics.

[53] Fellers R.T., Greer J.B., Early B.P., Yu X., LeDuc R.D., Kelleher N.L., Thomas P.M. (2015). ProSight lite: Graphical software to analyze top-down mass spectrometry data. Proteomics.

[54] Casadonte R., Caprioli R.M. (2012). Proteomic analysis of formalin-fixed paraffin embedded tissue by MALDI imaging mass spectrometry. Nat. Protoc..

[55] Kriegsmann J., Kriegsmann M., Casadonte R. (2015). MALDI TOF imaging mass spectrometry in clinical pathology: A valuable tool for cancer diagnostics (review). Int. J. Oncol..

[56] Kohn E.C., Azad N., Annunziata C., Dhamoon A.S., Whiteley G. (2007). Proteomics as a tool for biomarker discovery. Dis. Markers.

[57] Merlos Rodrigo M.A., Zitka O., Krizkova S., Moulick A., Adam V., Kizek R. (2014). MALDI-TOF MS as evolving cancer diagnostic tool: A review. J. Pharm. Biomed. Anal..

