# Supplementary material for: Mass Spectrometry Imaging Reveals Neutrophil Defensins as Additional Biomarkers for Anti-PD-(L)1 Immunotherapy Response in NSCLC Patients
Source: Cancers (Basel). 2020 Apr 2;12(4):863. doi: 10.3390/cancers12040863 (PMC7225984; doi:10.3390/cancers12040863)
Supplement: Supplementary file 1 [file cancers-12-00863-s001.pdf]

Mass Spectrometry Imaging Reveals Neutrophil Defensins as Additional Biomarkers for Anti-PD-(L)1 Immunotherapy Response in NSCLC Patients

Eline Berghmans [1,2], Julie Jacobs [3,4], Christophe Deben [3,4], Christophe Hermans [3,4], Glenn Broeckx [3,4], Evelien Smits [3,5], Evelyne Maes [6], Jo Raskin [7], Patrick Pauwels [3,4] and Geert Baggerman [1,2,*]

1 Centre for Proteomics, University of Antwerp, Antwerpen 2020, Belgium; eline.berghmans@vito.be
2 Health Unit, VITO, Mol 2400, Belgium
3 Center for Oncological Research, University of Antwerp, Wilrijk 2610, Belgium; julie.jacobs@uantwerpen.be (J.J.); christophe.deben@uantwerpen.be (C.D.); christophe.hermans@uantwerpen.be (C.H.); glenn.broeckx@uza.be (G.Br.); evelien.smits@uza.be (E.S.); patrick.pauwels@uza.be (P.P.)
4 Pathology Department, Antwerp University Hospital, Edegem 2650, Belgium
5 Center for Cell Therapy and Regenerative Medicine, Antwerp University Hospital, Edegem 2650, Belgium.
6 Food & Bio-Based Products, AgResearch Ltd., Lincoln 7674, New Zealand; evelyne.maes@agresearch.co.nz
7 Thoracic Oncology Department, Antwerp University Hospital, Edegem 2650, Belgium; jo.raskin@uza.be
* Correspondence: Geert.Baggerman@vito.be; Tel.: +32-476-472-918

Supplemtentary

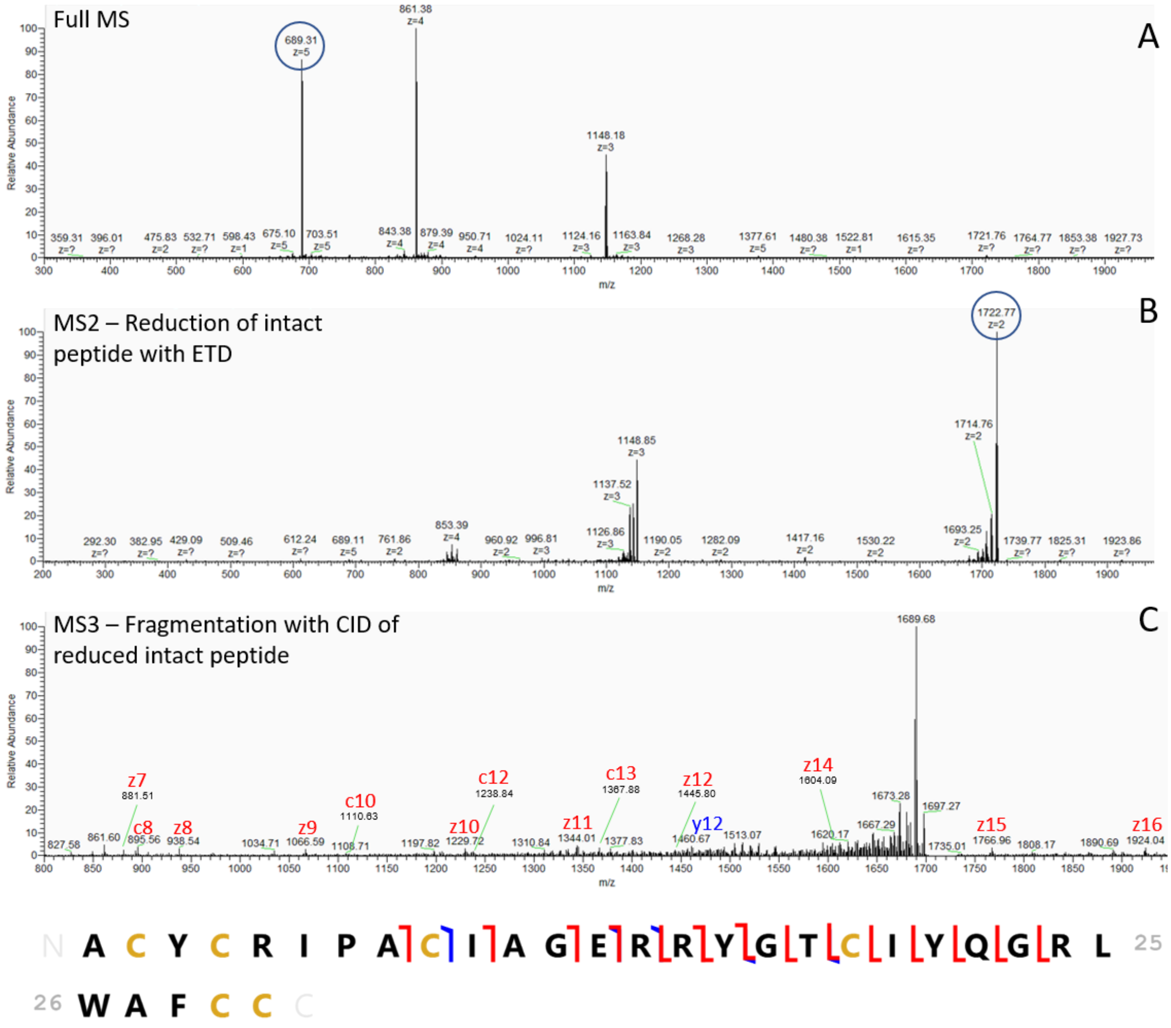

Figure S1. Mass spectra and annotated sequence of synthetic peptide corresponding for neutrophil defensin 1. (**A**) Full MS spectrum of intact neutrophil defensin 1, in three different charge states. The fifth charged ion with m/z 689.31 (mass 3,441.6 Da) is selected for reduction of the three internal disulfide

bridges with ETD (activation time 200 ms); (**B**) The resulting intact peptide m/z 1722.77 has lost three charges, one for reducing each disulfide bridge. This reduced peptide is immediately selected for fragmentation with CID (activation energy 45 for 70 ms); (**C**) The resulting fragmentation spectrum with b, c, y and z type ions. The main important ions are depicted on the fragmentation spectrum to confirm the identification of neutrophil defensin 1 (same ions, except for y9, y16 and c17, are detected in Figure 2B); (**D**) Annotated sequence of neutrophil defensin 1 with all matched deconvoluted fragments (see also Table S1) (b and y ions are indicated in blue, while c and z ions are indicated in red), obtained with both CID and ETD as fragmentation method.

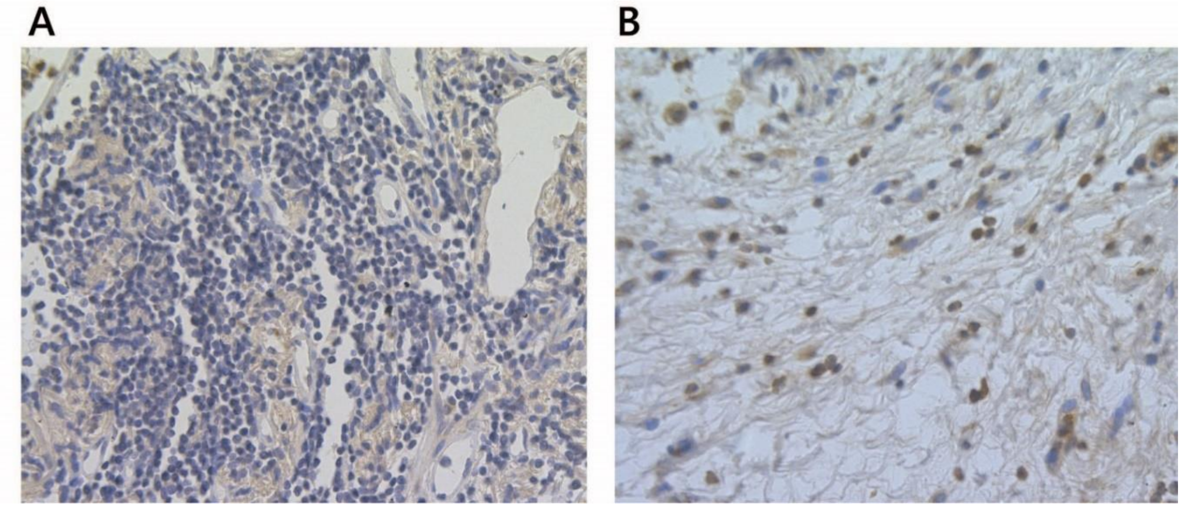

Figure S2. Examples of immunohistochemistry (IHC) results for defensin 1/3 antibody staining. (**A**) Example of defensin 1/3 IHC negative case; (**B**) Example of defensin 1/3 IHC positive case. Magnification 400×.

A

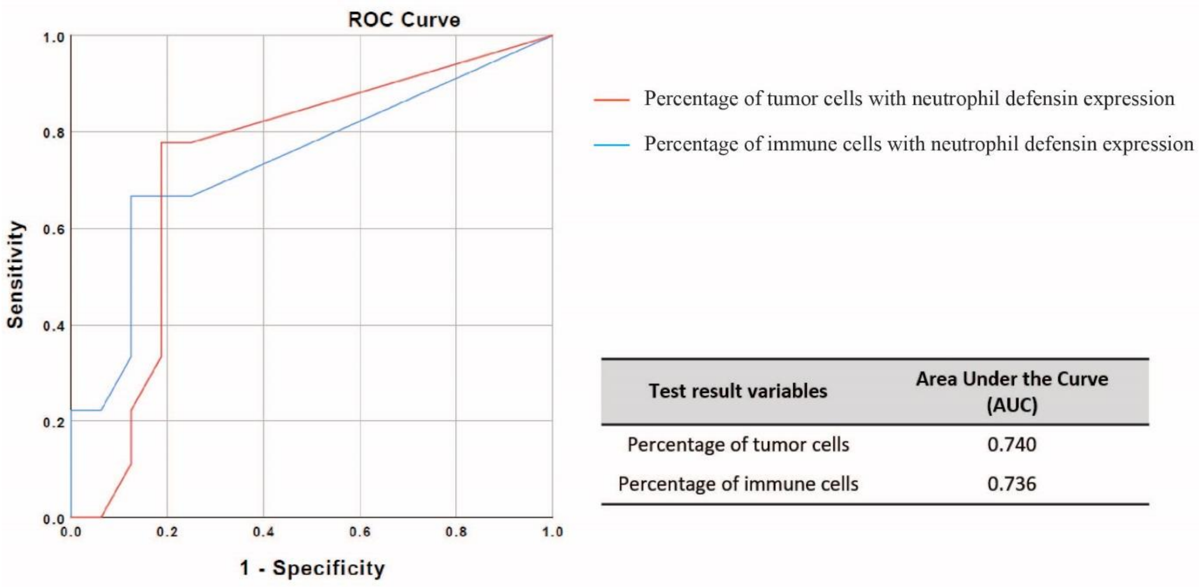

Test result variables	Area Under the Curve (AUC)
Percentage of tumor cells	0.740
Percentage of immune cells	0.736

B

Test result variable	Positive if greater than or equal to	Sensitivity	1 - Specificity
Percentage of tumor cells	-1.0000	1.000	1.000
	0.7500	0.667	0.250
	1.7500	0.667	0.125
	2.7500	0.444	0.125
	4.2500	0.333	0.125
	7.5000	0.222	0.063
	11.2500	0.222	0.000
	18.7500	0.111	0.000
	26.0000	0.000	0.000

Test result variable	Positive if greater than or equal to	Sensitivity	1 - Specificity
Percentage of immune cells	-1.0000	1.000	1.000
	0.7500	0.778	0.250
	1.7500	0.778	0.188
	3.5000	0.667	0.188
	6.2500	0.444	0.188
	8.7500	0.333	0.188
	12.5000	0.222	0.125
	25.0000	0.111	0.125
	42.5000	0.000	0.063
	51.0000	0.000	0.000

Figure S3. Receiver operating characteristic (ROC) curve for neutrophil defensin expression on tumor cells and immune cells within responders and nonresponders to immunotherapy. (**A**) ROC curve and corresponding Area Under the Curve (AUC); (**B**) Sensitivity and specificity for the detection of responders. The optimal was obtained with a cut-off of 1.75% for both cells, indicated in red.

Table S1. List of annotated fragment ions in the deconvoluted fragmentation spectrum of the synthetic peptide corresponding neutrophil defensin 1. The mixture of b, c, y and z fragment ions were aligned to the amino acid sequence of neutrophil defensin 1, generated with Prosight lite with 300 ppm mass accuracy.

Neutrophil Defensin 1 (Synthetic peptide) ACYCRIPACIAGERRYGTCIYQGRLWAFCC m/z 688.91; *z* = 5; Mass 3,439.55 Da Retention time 34.63 min						
Name	Ion Type	Ion Number	Theoretical Mass	Observed Mass	Mass Difference (Da)	Mass Difference (ppm)
b9	b	9	980.40305	980.520996	0.117946	120.3035833
b13	b	13	1350.58827	1350.891846	0.303576	224.773165
b14	b	14	1506.68938	1507.092285	0.402905	267.4107917
c8	c	8	894.42016	894.485413	0.065253	72.95564536
c10	c	10	1110.51341	1110.625122	0.111712	100.5949131
c12	c	12	1238.57198	1238.836182	0.264202	213.3117851
c13	c	13	1367.61457	1367.882813	0.268243	196.139326

c15	c	15	1679.81679	1680.310791	0.494001	294.0802848
c16	c	16	1842.88012	1843.172852	0.292732	158.8448412
y12	y	12	1461.635565	1461.773926	0.138361	94.66176338
y14	y	14	1619.704705	1620.173828	0.469123	289.6348937
z7	z	7	881.38018	881.512634	0.132454	150.2802117
z8	z	8	938.40164	938.541504	0.139864	149.0449228
z9	z	9	1066.46022	1066.590332	0.130112	122.003613
z10	z	10	1229.52355	1229.718994	0.195444	158.959135
z11	z	11	1342.60761	1342.889893	0.282283	210.2498138
z12	z	12	1445.6168	1445.802612	0.185812	128.5347542
z13	z	13	1546.66448	1546.984253	0.319773	206.7500768
z14	z	14	1603.68594	1604.087402	0.401462	250.3370454
z15	z	15	1766.74927	1766.95752	0.20825	117.8718472
z16	z	16	1922.85038	1923.041992	0.191612	99.64997901

Table S2. List of annotated fragment ions in the deconvoluted fragmentation spectrum. The mixture of c, y and z fragment ions were aligned to the amino acid sequence of neutrophil defensin 2, generated with Prosight lite with 300 ppm mass accuracy.

Neutrophil Defensin 2
CYCRIPACIAGERRYGTCIYQGRLWAFCC
m/z 674.70; *z* = 5; Mass 3368.50 Da
Retention time 32.42 min

Name	Ion Type	Ion Number	Theoretical Mass	Observed Mass	Mass Difference (Da)	Mass Difference (ppm)
c7	c	7	823.38305	823.594604	0.211554	256.9326634
c12	c	12	1296.57746	1296.84729	0.26983	208.109433
c13	c	13	1452.67857	1452.891113	0.212543	146.3111003
c14	c	14	1608.77968	1609.172974	0.393294	244.4672847
y11	y	11	1358.626375	1358.781738	0.155363	114.3529986
y13	y	13	1562.683245	1563.130005	0.44676	285.8928714
z7	z	7	881.38018	881.598877	0.218697	248.1301542
z8	z	8	938.40164	938.545715	0.144075	153.5323404
z9	z	9	1066.46022	1066.759644	0.299424	280.7643402
z10	z	10	1229.52355	1229.793579	0.270029	219.6208442
z11	z	11	1342.60761	1342.879272	0.271662	202.3390885
z12	z	12	1445.6168	1445.903931	0.287131	198.6217924
z13	z	13	1546.66448	1546.879395	0.214915	138.9538602
z14	z	14	1603.68594	1603.933228	0.247288	154.1997681
z15	z	15	1766.74927	1767.046387	0.297117	168.1715708
z16	z	16	1922.85038	1923.084229	0.233849	121.6158066

Table 3. List of annotated fragment ions in the deconvoluted fragmentation spectrum of the synthetic peptide corresponding neutrophil defensin 2. The mixture of b, c, y and z fragment ions were aligned to the amino acid sequence of neutrophil defensin 2, generated with Prosight lite with 300 ppm mass accuracy.

Neutrophil Defensin 2 (Synthetic peptide)
CYCRIPACIAGERRYGTCIYQGRLWAFCC
m/z 674.70; *z* = 5; Mass 3,368.50 Da
Retention time 33.75 min

Name	Ion Type	Ion Number	Theoretical Mass	Observed Mass	Mass Difference (Da)	Mass Difference (ppm)
b9	b	9	1022.45	1022.730652	0.280652	274.4897061
b12	b	12	1279.55116	1279.786865	0.235705	184.2091253
b13	b	13	1435.65227	1435.666992	0.014722	10.2545723
b16	b	16	1811.83817	1812.239746	0.401576	221.6401038
c7	c	7	823.38305	823.495239	0.112189	136.2537157
c8	c	8	926.39224	926.587891	0.195651	211.1967173
c9	c	9	1039.4763	1039.71106	0.23476	225.844495
c10	c	10	1110.51341	1110.563232	0.049822	44.8639337
c11	c	11	1167.53487	1167.722778	0.187908	160.9442294
c12	c	12	1296.57746	1296.859619	0.282159	217.6183134

c13	c	13	1452.67857	1453.050049	0.371479	255.7200248
c16	c	16	1828.86447	1829.104004	0.239534	130.9741667
c17	c	17	1929.91215	1930.213867	0.301717	156.337168
y7	y	7	897.398945	897.468018	0.069073	76.97022644
y9	y	9	1082.478985	1082.581665	0.10268	94.85634495
y12	y	12	1461.635565	1461.616943	−0.018622	−12.74052195
y15	y	15	1782.768035	1782.976563	0.208528	116.9686666
y16	y	16	1938.869145	1939.165283	0.296138	152.7374866
z7	z	7	881.38018	881.513794	0.133614	151.5963293
z8	z	8	938.40164	938.54834	0.1467	156.3296501
z9	z	9	1066.46022	1066.575928	0.115708	108.497249
z10	z	10	1229.52355	1229.704102	0.180552	146.8471263
z11	z	11	1342.60761	1342.837646	0.230036	171.3352422
z12	z	12	1445.6168	1445.892822	0.276022	190.9371834
z13	z	13	1546.66448	1546.772705	0.108225	69.97315927
z14	z	14	1603.68594	1603.699707	0.013767	8.584598553
z15	z	15	1766.74927	1766.914307	0.165037	93.41280215
z16	z	16	1922.85038	1923.000977	0.150597	78.31966624

Table S4. List of annotated fragment ions in the deconvoluted fragmentation spectrum. The mixture of b, c, y and z fragment ions were aligned to the amino acid sequence of neutrophil defensin 3, generated with Prosight lite with 300 ppm mass accuracy.

Neutrophil Defensin 3<br>DCYCRIPACIAGERRYGTCIYQGRLWAFCC<br>m/z 697.71; *z* = 5; Mass 3,483.55 Da<br>Retention time 32.21 min						
**Name**	**Ion Type**	**Ion Number**	**Theoretical Mass**	**Observed Mass**	**Mass Difference (Da)**	**Mass Difference (ppm)**
b15	b	15	1706.78032	1706.319824	−0.460496	−269.8039077
b15	b	15	1706.78032	1707.215088	0.434768	254.7299116
b17	b	17	1926.86511	1927.2146	0.34949	181.3775122
c8	c	8	938.40999	938.541016	0.131026	139.6255383
c11	c	11	1225.54035	1225.755737	0.215387	175.7485994
c12	c	12	1282.56181	1282.87561	0.3138	244.6665709
c13	c	13	1411.6044	1411.87915	0.27475	194.6366843
c14	c	14	1567.70551	1568.07251	0.367	234.1000894
y11	y	11	1358.626375	1358.895508	0.269133	198.0919883
y12	y	12	1461.635565	1461.801147	0.165582	113.2854208
y13	y	13	1562.683245	1563.066528	0.383283	245.2723552
y14	y	14	1619.704705	1619.254883	−0.449822	−277.718524
y14	y	14	1619.704705	1620.119751	0.415046	256.2479437
z7	z	7	881.38018	881.528442	0.148262	168.2157182
z8	z	8	938.40164	938.541016	0.139376	148.5248896
z9	z	9	1066.46022	1066.593506	0.133286	124.9798141
z10	z	10	1229.52355	1229.714233	0.190683	155.0869034
z11	z	11	1342.60761	1342.716797	0.109187	81.32458001
z12	z	12	1445.6168	1445.846436	0.229636	158.8498418
z13	z	13	1546.66448	1546.86499	0.20051	129.6402695
z14	z	14	1603.68594	1603.986938	0.300998	187.6913631
z15	z	15	1766.74927	1767.060303	0.311033	176.0481837
z16	z	16	1922.85038	1922.958496	0.108116	56.22694367

Table S5. List of annotated fragment ions in the deconvoluted fragmentation spectrum of the synthetic peptide corresponding neutrophil defensin 3. The mixture of b, c, y and z fragment ions were aligned to the amino acid sequence of neutrophil defensin 3, generated with Prosight lite with 300 ppm mass accuracy.

Neutrophil Defensin 3 (Synthetic peptide)<br>DCYCRIPACIAGERRYGTCIYQGRLWAFCC<br>m/z 697.72; *z* = 5; Mass 3,483.60 Da<br>Retention time 35.01 min						
**Name**	**Ion Type**	**Ion Number**	**Theoretical Mass**	**Observed Mass**	**Mass Difference (Da)**	**Mass Difference (ppm)**
b7	b	7	850.34658	850.575562	0.228982	269.2807914
b8	b	8	921.38369	921.58252	0.19883	215.7950072
b9	b	9	1024.39288	1024.611694	0.218814	213.6035932
b12	b	12	1265.53551	1265.582031	0.046521	36.75993256
b13	b	13	1394.5781	1394.901245	0.323145	231.7152406
b15	b	15	1706.78032	1706.718018	−0.062302	−36.50264728
b17	b	17	1926.86511	1927.193359	0.328249	170.3539071
c8	c	8	938.40999	938.539001	0.129011	137.4782892
c9	c	9	1041.41918	1041.654907	0.235727	226.3516983
c10	c	10	1154.50324	1154.626831	0.123591	107.051237
c11	c	11	1225.54035	1225.699951	0.159601	130.2290863
c12	c	12	1282.56181	1282.857056	0.295246	230.2002116
c13	c	13	1411.6044	1411.762451	0.158051	111.9655053
c14	c	14	1567.70551	1567.966064	0.260554	166.2008575
y7	y	7	897.398945	897.577209	0.178264	198.6452079
y9	y	9	1082.478985	1082.591309	0.112324	103.7655248
y11	y	11	1358.626375	1358.992432	0.366057	269.4316898
y12	y	12	1461.635565	1461.834351	0.198786	136.0024378
y13	y	13	1562.683245	1562.249268	−0.433977	−277.7127107
y13	y	13	1562.683245	1563.052124	0.368879	236.0548762
y14	y	14	1619.704705	1619.233643	−0.471062	−290.8320255
y15	y	15	1782.768035	1783.074707	0.306672	172.0201361
y16	y	16	1938.869145	1939.262939	0.393794	203.1049909
z7	z	7	881.38018	881.545898	0.165718	188.0210195
z8	z	8	938.40164	938.539001	0.137361	146.3776214
z9	z	9	1066.46022	1066.564819	0.104599	98.08054538
z10	z	10	1229.52355	1229.748779	0.225229	183.1839659
z11	z	11	1342.60761	1342.807251	0.199641	148.6964609
z12	z	12	1445.6168	1445.784302	0.167502	115.8688803
z13	z	13	1546.66448	1547.004395	0.339915	219.77294
z14	z	14	1603.68594	1603.870361	0.184421	114.9982022
z15	z	15	1766.74927	1766.998535	0.249265	141.0867995
z16	z	16	1922.85038	1923.01001	0.15963	83.01737965
